# Iodine adequacy in reproductive age and pregnant women living in the Western region of Saudi Arabia

**DOI:** 10.1186/s12884-020-03057-w

**Published:** 2020-06-22

**Authors:** Firas Azzeh, Bassem Refaat

**Affiliations:** 1 Clinical Nutrition Department, Faculty of Applied Medical Sciences, Umm Al-Qura University, Al Abdeyah, PO Box 7607, Makkah, Saudi Arabia; 2 Laboratory Medicine Department, Faculty of Applied Medical Sciences, Umm Al-Qura University, Al Abdeyah, Holy Makkah, PO Box 7607, Makkah, Saudi Arabia

**Keywords:** Iodine supplement, Iodine-measurement, Pregnancy and nutrition

## Abstract

**Background:**

Despite the serious maternal and foetal complications associated with iodine deficiency during pregnancy, surveys related to pregnant women in the Kingdom of Saudi Arabia (KSA) are lacking. This study, therefore, measured urine iodine concentrations (UIC) alongside the potential socioeconomic factors contributing towards iodine inadequacy in reproductive age and pregnant Saudi women from the Western province of KSA.

**Methods:**

Spot urine samples were collected from 1222 pregnant and 400 age-matched non-pregnant/non-lactating reproductive age women. The socioeconomic characteristics were obtained through a structured questionnaire. The WHO criteria for iodine sufficiency in non-pregnant (100–199 μg/L) and pregnant (150–249 μg/L) women were applied.

**Results:**

The median UIC in the non-pregnant women (101.64 μg/L; IQR: 69.83–143.55) was at the lowermost WHO recommended cut-off, whereas the pregnant group was iodine deficient (112.99 μg/L; IQR: 81.01–185.57). Moreover, the median UIC was below adequacy across the different trimesters. The use of non-iodised salt significantly increased the risk of iodine deficiency in the non-pregnant (OR = 2.052; 95%CI: 1.118–3.766) and pregnant women (OR = 3.813; 95%CI: 1.992–7.297), whereas taking iodine supplements significantly lowered the risk in both groups (OR = 0.364; 95%CI: 0.172–0.771 and OR = 0.002; 95%CI: 0.001–0.005, respectively). Passive smoking was also an independent risk factor for iodine deficiency in the non-pregnant (OR = 1.818; 95%CI: 1.097–3.014) and pregnant (OR = 1.653; 95%CI: 1.043–2.618) groups. Additionally, BMI correlated independently and significantly with median UIC in the non-pregnant and pregnant populations. However, multiparity (OR = 3.091; 95%CI: 1.707–5.598) and earning below the minimum wage (2.520; 95%CI: 1.038–6.119) significantly increased the risk of iodine deficiency only in the non-pregnant women.

**Conclusions:**

This study is the first to show borderline iodine sufficiency in reproductive age Saudi women from the Western province, whereas mild iodine deficiency was observed in the pregnant population and could represent a serious public health problem. This study also advocates the necessity to establish routine iodine dietary advice services by the health authorities to foster adequate iodine intake in pregnant women to avoid the perilous consequences of iodine deficiency on maternal-foetal health.

## Background

Iodine is essential for the synthesis of thyroid hormones, and suboptimal intake of this nutrient at the periconceptual and/or during pregnancy has been linked with maternal goitre and hypothyroidism [1, 2]. Iodine deficiency during pregnancy also significantly increases the risk of delayed neurodevelopment and poor cognitive functions of the offspring [3, 4]. Although many countries have implemented the universal salt iodisation (USI) policy to ensure adequate access to iodine by the general public [5, 6], numerous reports from developed and developing countries have demonstrated high rates of iodine insufficiency among pregnant women [7–11]. Accordingly, many researchers have emphasised the need for alternative means (e.g. iodine supplements) to ensure adequate iodine intake during pregnancy [7–11], and the World Health Organisation (WHO) has recommended that the urine iodine concentrations (UIC) should range between 150 to 250 μg/L to assure adequacy during pregnancy [12].

About 90% of ingested iodine from diet and/or supplement is excreted by the kidney and, measuring UIC in spot samples and estimating 24-h urine iodine excretion (24-h UIE) are reliable approaches for assessing iodine status [13–15]. Surveying school-age children (SAC) is also the currently accepted method for evaluating iodine intake within a population since they are easy to access and are believed to reflect the nutritional status of their families [16]. However, the most recent report by the Iodine Global Network (IGN) in 2019 has revealed that 29 countries reported iodine deficiency in pregnant women albeit that their SAC populations were sufficient [5]. Hence, it has been proposed that pregnant women should be surveyed independently from SAC to precisely measure their iodine status [7–11]. Additionally, the updated guidelines by the United Nations International Children’s Emergency Fund (UNICEF) has also recommended that iodine should be assessed in different subsets of a population, especially those who are vulnerable for deficiency [17].

In the Kingdom of Saudi Arabia (KSA), thyroid disorders are common among the general population at the different ages [18–21] and goitre was frequently reported in Saudi children, particularly among those who were living in high altitude areas of the kingdom [22, 23]. Alissa et al. (2009) followed the WHO recommended median UIC cut-off values for iodine adequacy, and their results revealed severe deficiency in their enrolled hypothyroid patients as well as healthy participants from the Western region of KSA [24]. Although KSA is currently classified by the IGN (2019) as iodine sufficient based on a national survey of SAC in 2012 [5], more recent national studies have revealed marked iodine deficiency and high prevalence of goitre in their SAC populations [25, 26]. According to another recent national survey, KSA has also adopted the USI policies since 1995 and most of the locally available salt is adequately iodised (15–40 ppm) [27]. However, salt iodisation is not obligatory in the kingdom, only 70% of the Saudi households were found to consume iodised salt, and the numbers are below the WHO references [27]. Concomitantly, we have previously shown that 26.8 and 4.8% of pregnant Saudi women from the Western region had hypothyroidism and isolated hypothyroxinaemia, respectively [28]. However, currently there is no report on iodine status among reproductive age and pregnant Saudi women.

Hence, this study measured iodine adequacy in non-pregnant, non-lactating reproductive age Saudi women as well as in pregnant Saudi women at the different trimesters. Additionally, the socio-economic characteristics were collected to identify the factors that could contribute to iodine deficiency. A better understanding about iodine status in the targeted populations could enlighten the health authorities and policymakers regarding the magnitude of iodine deficiency and may possibly support the development of appropriate policies regarding the screening and prevention of iodine inadequacy during pregnancy.

## Methods

### Study design

This non-randomised, cross-sectional study was conducted from March 2017 to May 2019, and ethical approval (AMSEC 21–16-02-2017) was obtained from the Faculty of Applied Medical Sciences Ethics Committee in Umm Al-Qura University. The study populations included 1222 apparently healthy pregnant Saudi women (18–44 years) at the different trimesters and who were recruited from the antenatal care unit in the Medical Centre of Umm Al-Qura University in Makkah City. Another 400 primi- or multiparous non-pregnant, non-lactating, reproductive age Saudi women, and who had no relevant bad obstetrics history were also recruited from the same centre during the routine vaccination of their children. All the pregnant and non-pregnant participants had no current symptoms/signs or history of thyroid disorders, chronic diseases (e.g. hypertension, diabetes mellitus, etc.), gestational related medical disorders (e.g. pre-eclampsia, gestational diabetes, anaemia, etc.) or autoimmune diseases. According to the 2017 reports issued by the Saudi General Authority for Statistics and Ministry of Health, the total numbers of reproductive age and pregnant Saudi women in the Western region of KSA were 1,140,185 and 29,701, respectively [29, 30]. The Epi Info™ software was used (https://www.cdc.gov/epiinfo/index.html), and the minimal required sample sizes were 384 non-pregnant and 379 pregnant women to achieve a study power of 95%.

A fresh spot urine sample (5 ml) was collected from each woman in a sterile urine container between 9:00 am and 1:00 pm and the samples were stored at − 70 °C till transported to the Research Laboratories of the Faculty of Applied Medical Sciences in Umm Al-Qura University for processing. Additionally, the socioeconomic characteristics were obtained through a structured questionnaire that included information related to age (years), pre-pregnancy and/or current weight (Kg) and height (cm) to calculate the body mass index (BMI), parity, type of salt intake, the use of daily iodine-containing supplements, family size, education level, employment status, total monthly income, smoking, residency and gestational age at the time of sample collection from the pregnant population. The question related to the use of iodised salt was categorised as previously reported into non-iodised, iodised and I don’t know [31].

### Urine Creatinine concentrations

Urine creatinine concentrations were measured on Cobas e411 (Roche Diagnostics International Ltd.; Risch-Rotkreuz, Switzerland) according to the manufacturer’s protocol. The predicted 24-h urine creatinine excretion (24-h Cr; g/day) was calculated by the previously published equation as follow [32]:

Predicted 24-h Cr for females = (0.00163 × [140 - age (years)] × [weight (kg)^1.5^ × height (cm)^0.5^] × [1 + 0.18 × (black = 1, nonblack = 0)] × [1.429–0.0198 × BMI (kg/m^2^)]/1000).

### Urine iodine concentrations

All the chemicals were analytical grade from Sigma-Aldrich Co. (MO, USA) and the required working solutions for measuring urine iodine were prepared as previously described [15]. The spot urine samples were processed in 96-well clear polystyrene flat-bottomed microplates (Thermo Fisher Scientific, CA, USA) and on a fully automated ELISA machine (Human Diagnostics, Wiesbaden, Germany) to measure the UIC according to the principles of the Sandell-Kolthoff method [15]. Low (13 μg/l), intermediate (102 μg/l) and high (203 μg/l) iodine calibrators were also processed five times to measure the assay performances. The intra-assay coefficient of variation was 5.7, 4.3 and 4.1% for the low, intermediate and high calibrators, respectively. Additionally, the inter-assay precision was 10% for the low, 7% for the intermediate and 4.5% for the high calibrators.

The 24-h UIE was calculated according to the previously published equation as follow [14]: Spot urine [Iodine/Creatinine (μg/g)] × predicted 24-h Cr (g/day). The amount of 24-h UIE were classified according to the WHO reference values for iodine adequacy as follow: deficiency (< 100 and <  150 μg/day), sufficiency (100–199 and 150–249 μg/day) and excess (≥ 200 and ≥ 250 μg/day) for non-pregnant and pregnant women, respectively [13].

### Statistical analysis

Statistical analysis was done with SPSS software version 25 (New York, USA), and *P* <  0.05 was considered statistically significant. The Kolmogorov-Smirnov test for normality and Levene test for homogeneity were performed for continuous data that were expressed either as mean ± standard deviation (SD) or median with the interquartile range (IQR) depending on data normality. Ordinal and discontinuous data were shown as numbers and percentages, and cross-tabulation followed by Chi square (χ^2^) test were applied for frequency analysis. Based on data normality, one-way ANOVA or Kruskal–Wallis were used to compare between more than two groups followed by either Tukey’s HSD or Games-Howell post-hoc tests according to variance equality. Multinomial regression analysis was also performed to identify the socioeconomic predictors of iodine status in each of the study populations.

## Results

### The socioeconomic characteristics of the study groups

The study raw data are available as Supplementary File 1. The mean ± SD of age and BMI in the non-pregnant population were 29.1 ± 7.3 years and 24.03 ± 5.4 kg/m^2^, respectively. The majority of participants were primiparous (*n* = 228; 57%) and their families comprised ≤ four members (*n* = 236; 59%). Nineteen (4.7%) women were illiterate, whereas 34 (8.5%) had primary education, 125 (31.2%) had secondary education, and the remaining 222 subjects (55.5%) had a university degree. However, unemployment was predominant (*n* = 311; 77.8%). While 14% (*n* = 56) reported that their monthly income was below the Saudi national minimum wage (3000 SAR), 37.3% (*n* = 159) were receiving between 3001 and 5000 SAR, 33.5% (*n* = 134) were gaining between 5001 and below 10,000 SAR and 15.3% (*n* = 61) were earning ≥10,000 SAR. Active and passive smoking were also reported by 5.8% (*n* = 23) and 34.2% (*n* = 137), respectively. Moreover, 292 women (73%) were using iodised salt and the remainders either were using non-iodised salt (*n* = 84; 21%) or chose ‘I don’t know’ (*n* = 24; 6%). Finally, 46 women (11.5%) were using daily iodine supplements (150 μg/day). The socio-economic characteristics of the non-pregnant population are detailed in Supplementary File 2.

On the other hand, the mean of age was 28.7 ± 5.7 years and the mean of pre-conceptional BMI was 25.7 ± 4.7 kg/m^2^ in the pregnant group (*n* = 1222). Multigravidity (*n* = 956; 78.2%), having more than four family members (*n* = 786; 64.3%), being a university graduate (*n* = 694; 56.8%), unemployment (n = 956; 78.2%) and earning a monthly income between 3001 and 5000 SAR (*n* = 642; 52.5%) were common among the pregnant population. Furthermore, the majority was neither active (*n* = 1158; 94.8%) nor passive (*n* = 792; 64.8%) smokers. Meanwhile, 71.5% (*n* = 874) were using iodised salt, 21.8% (*n* = 266) were utilising non-iodised salt and 6.7% (*n* = 82) answered by ‘I don’t know’. Additionally, only 338 (27.7%) pregnant women reported using iodine supplements (220 μg/day). The sub-analysis also showed that 364 (29.8%), 352 (28.8%) and 506 (41.4%) women were in the first, second and third trimesters, respectively. Furthermore, the distribution of age, BMI, family size, educational levels, monthly income, active smoking, the use of iodised salt and consumption of iodine supplements were significantly different between the three trimesters (Table 1).

**Table 1:** The socioeconomic characteristics of the pregnant participants (*n* = 1222)

**Parameter**	**Pregnant group** **(*n* = 1222)**			
	***First trimester*** ***(n = 364; 29.8%)***	***Second trimester*** ***(n = 352; 28.8%)***	***Third trimester*** ***(n = 506; 41.4%)***	***P-value***
***Mean ± SD of Age (year)***	28.6 ± 6.2	27.9 ± 5.5	29.2 ± 5.4	**0.003**
***Age groups***				**< 0.001**
18- < 25	132 (10.8%)	134 (10.9%)	118 (9.7%)	
25- < 35	160 (13.1%)	172 (14.1%)	296 (24.2%)	
> 35	72 (5.9%)	46 (3.8%)	92 (7.5%)	
***Mean ± SD of Weight (kg)***	66.4 ± 13.6	65.9 ± 13.5	69.1 ± 13.4	**0.001**
***Mean ± SD of Height (cm)***	160.8 ± 7.9	160.9 ± 7.03	163.3 ± 8.4	**< 0.001**
***Mean ± SD of BMI (kg/m***^***2***^***)***	25.8 ± 5.5	25.4 ± 4.6	25.8 ± 4.3	0.3
***BMI Classes***				**0.006**
Underweight	12 (1%)	18 (1.5%)	24 (2%)	
Normal	164 (13.4%)	156 (12.8%)	186 (15.2%)	
Overweight	124 (10.2%)	136 (11.1%)	230 (18.8%)	
Obese	64 (5.2%)	42 (3.4%)	66 (5.4%)	
***Parity***				0.1
Primiparous	72 (5.9%)	90 (7.4%)	104 (8.5%)	
Multiparous	292 (23.9%)	262 (21.4%)	402 (32.9%)	
***Family Size***				**0.02**
≤ 4 members	240 (19.7%)	248 (20.3%)	298 (24.4%)	
> 4 members	124 (10.1%)	104 (8.5%)	208 (12.8%)	
***Total income (SR)***				0.08
< 3000	78 (6.4%)	64 (5.2%)	123 (10.1%)	
3001–5000	196 (16%)	196 (16%)	250 (20.4%)	
5001–10,000	76 (6.2%)	68 (5.6%)	114 (9.3%)	
> 10,001	14 (1.2%)	24 (2%)	19 (1.6%)	
***Education Level***				**< 0.001**
Illiterate	16 (1.3%)	20 (1.6%)	40 (3.3%)	
1^ry^ Education	32 (2.6%)	26 (2.1%)	52 (4.2%)	
2^ry^ Education	76 (6.2%)	94 (7.7%)	172 (14.1%)	
University	240 (19.7%)	212 (17.4%)	242 (19.8%)	
***Employment***				0.1
Yes	92 (7.5%)	74 (6.1%)	100 (8.2%)	
No	272 (22.3%)	278 (22.7%)	406 (33.2%)	
***Residency***				0.8
Urban	356 (29.1%)	342 (28%)	494 (40.4%)	
Rural	8 (0.7%)	10 (0.8%)	12 (1%)	
***Active smoking***				**0.004**
Yes	8 (0.7%)	19 (1.6%)	37 (3%)	
No	356 (29.1%)	333 (27.2%)	469 (38.4%)	
***Passive smoking***				0.2
Yes	128 (10.5%)	112 (9.1%)	190 (15.5%)	
No	236 (19.3%)	240 (19.7%)	316 (25.9%)	
***Salt Intake***				**0.03**
Don’t know	28 (2.3%)	12 (1%)	42 (3.4%)	
Non-iodised	84 (6.9%)	82 (6.7%)	100 (8.2%)	
Iodised	252 (20.6%)	258 (21.1%)	364 (29.8%)	
***Iodine supplement***				**0.01**
Yes	80 (6.6%)	106 (8.7%)	152 (12.4%)	
No	284 (23.2%)	246 (20.1%)	354 (29%)	

### Urine iodine concentrations and associated factors with iodine inadequacy

#### A. Non-pregnant population

The median spot urine iodine and creatinine concentrations together with the I/Cr ratio in the entire non-pregnant population were 75.3 μg/L (IQR: 51.6–106.2), 0.73 g/L (IQR: 0.62–0.85) and 102.86 μg/g (IQR: 67.6–153.2), respectively. Additionally, the estimated 24-h urine Cr excretion in the non-pregnant women was 1.1 g/L (IQR: 0.97–1.32), whereas the 24-h UIE (101.64 μg/L; IQR: 69.83–143.55) was at the lowest WHO recommended limit for adequate iodine intake for reproductive age women (100–199 μg/L).

The sub-analysis showed that the group of non-pregnant women not using iodine supplements (*n* = 354; 88.5%) had significantly lower spot median UIC (70.5 μg/L; IQR: 49.1–96.1 vs. 120.3 μg/L; IQR: 70.4–134.4) and I/Cr ratio (98.6 μg/g; IQR: 65.8–144.1 vs. 170.2 μg/g; IQR: 116.4–226.6) compared with those who were taking supplements rich in iodine (*n* = 46; 11.5%). Furthermore, the 24-h UIE in the non-pregnant individuals not using iodine supplements (95.2 μg/L; IQR: 66.1–131.4) was also significantly lower than the subgroup using supplements (165.6 μg/L; IQR: 95.1–182.4), and the levels were below the WHO recommended levels. However, the median of spot Cr and estimated 24-h urine Cr excretion were comparable between both groups.

The regression analysis showed that having > 4 family members (2.4-fold), earning below the national minimal wage (2.5-fold), passive smoking (1.8-fold) and using non-iodised salt (2-fold) significantly increased the risk of iodine deficiency in the non-pregnant population (Table 2). Contrariwise, primiparity (3-fold) and the consumption of iodine supplements (2.7-fold) significantly decreased the risk of iodine deficiency. Furthermore, low educational level (illiterate; 10-fold and primary education; 4-fold) was the only independent factor that increased the risk of iodine intake above requirements (Table 2), whereas BMI was independently and significantly associated with median UIC in the non-pregnant women (1.084; 95%CI: 1.010–1.163).

**Table 2:** The socioeconomic risk factors associated with insufficient and excess iodine intake among the non-pregnant participants (*n* = 400) by multinomial regression analysis

**Risk factors**	**Insufficiency (< 100 μg/L)**		**Above requirements** **(≥ 200 μg/L)**	
	***Odds ratio (95%CI)***	***P Value***	***Odds ratio (95%CI)***	***P Value***
**Age (years)**	1.017 (0.983–1.052)	NS	0.978 (0.919–1.041)	NS
**BMI (Kg/m**^**2**^**)**	1.004 (0.959–1.051)	NS	1.084 (1.010–1.163)	***P = 0.02***
***Parity***				
Primiparous	**Ref.**		**Ref.**	
Multiparous	3.091 (1.707–5.598)	***P*** **< 0.0001**	0.607 (0.214–1.724)	NS
***Family Size***				
≤ 4 members	**Ref.**		**Ref.**	
> 4 members	2.390 (1.390–4.112)	***P*** **= 0.02**	2.102 (0.843–5.242)	NS
***Total income (SR)***				
< 3000	2.520 (1.038–6.119)	***P*** **= 0.04**	1.452 (0.333–6.338)	NS
3001–5000	1.465 (0.740–2.902)	NS	0.755 (0.219–2.595)	NS
5001–10,000	1.552 (0.769–3.012)	NS	0.707 (0.203–2.458)	NS
> 10,001	**Ref.**		**Ref.**	
***Education Level***				
Illiterate	1.387 (0.355–5.416)	NS	9.884 (2.028–48.163)	***P*** **< 0.01**
Primary Education	0.549 (0.224–1.344)	NS	4.212 (1.218–14.570)	***P = 0.02***
Secondary Education	1.087 (0.639–1.848)	NS	1.774 (0.662–4.596)	NS
University	**Ref.**		**Ref.**	
***Employment***				
Yes	1.009 (0.546–1.867)	NS	0.769 (0.273–2.165)	NS
No	**Ref.**		Ref.	
***Active smoking***				
Yes	1.226 (0.394–3.815)	NS	0.699 (0.069–7.043)	NS
No	**Ref.**		Ref.	
***Passive smoking***				
Yes	1.818 (1.097–3.014)	***P = 0.02***	1.309 (0.549–3.125)	NS
No	**Ref.**		Ref.	
***Salt Intake***				
Iodized	**Ref.**		**Ref.**	
Non-iodized	2.052 (1.118–3.766)	***P = 0.02***	1.247 (0.420–3.700)	NS
Don’t know	0.449 (0.161–1.251)	NS	0.753 (0.167–3.398)	NS
***Iodine supplement***				
Yes	0.364 (0.172–0.771)	***P < 0.01***	1.367 (0.479–3.900)	NS
No	**Ref.**			

*Ref* Reference category

*NS* Non-significant

#### B. Pregnant population

The spot urine concentrations in the overall pregnant population were 83.69 μg/L (IQR: 60–137.46) for UIC, 0.83 g/L (IQR: 0.71–0.97) for creatinine and 102.47 μg/g (IQR: 74.88–151.09) for I/Cr ratio. The estimated 24-h urine Cr median was 1.12 g/L (IQR: 0.97–1.32), whereas the 24-h UIE median (112.99 μg/L; QR: 81.01–185.57) was less than the WHO recommended minimal limit for adequate iodine intake during pregnancy (150–249 μg/L).

**Fig. 1:**
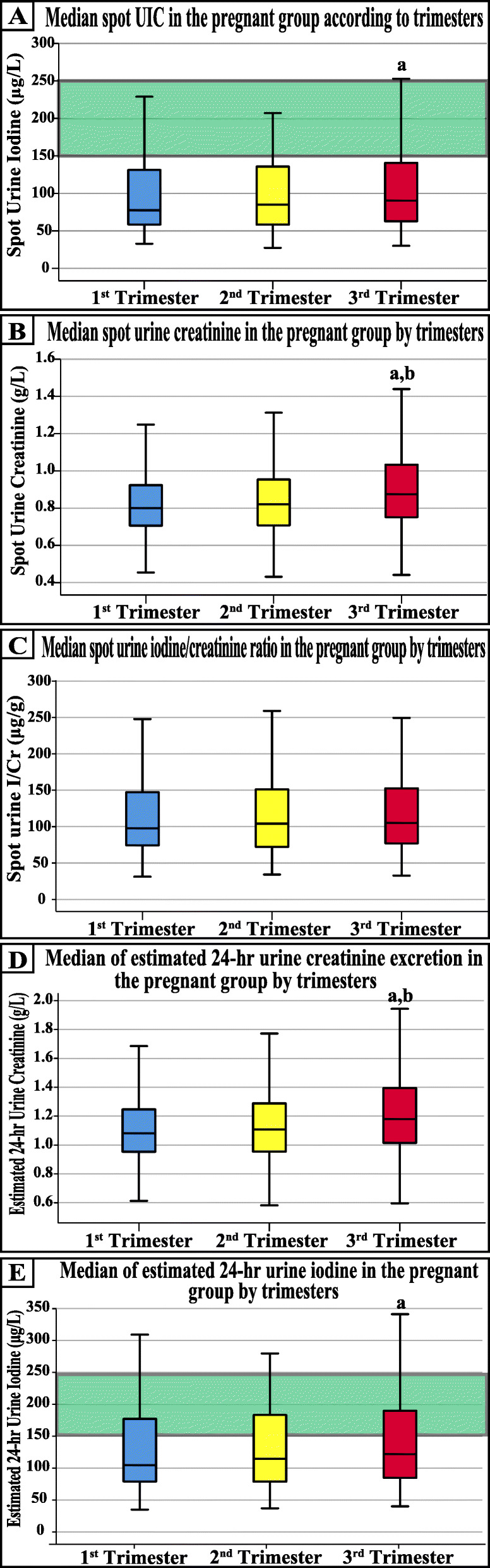
The median of (**a**) spot urine iodine concentrations, (**b**) spot urine creatinine concentrations, (**c**) urine spot iodine/creatinine ratio, (**d**) estimated 24-h urine creatinine excretion and (**e**) estimated 24-h urine iodine excretion in each trimester of pregnancy. (a = *P* <  0.05 compared with the 1st trimester and b = P <  0.05 compared with 2nd trimester by one-way ANOVA and green rectangle = the recommended WHO intervals for iodine adequacy in pregnant women)

While the first trimester median spot UIC was significantly lower than the third trimester (*P* = 0.02), the concentrations of both groups were comparable to the second trimester (Fig. 1a). Furthermore, the spot urine creatinine concentrations were similar in the first and second trimesters, and both groups were significantly lower than the third trimester levels (Fig. 1b). However, no significant difference was detected in the I/Cr ratio between the three trimesters (Fig. 1c). Although the estimated 24-h urine creatinine was also significantly lower in the first and second trimesters compared with the third trimester (Fig. 1d), the 24-h UIE was only significantly different between the first and third trimesters (Fig. 1e; *P* = 0.004). Nevertheless, the 24-h UIE levels in each of the three trimesters were below the WHO advocated minimum for adequate iodine during pregnancy (Fig. 1e).

By comparing between the pregnant women with (*n* = 338; 27.7%) and without (*n* = 884; 72.3%) iodine supplements, the median of spot UIC (70.1 μg/L; IQR: 53.1–90.3 vs. 152.3 μg/L; IQR: 138.1–166.9), Cr (0.79 g/L; IQR: 0.7–0.92 vs. 0.94 g/L; IQR: 0.81–1.1) and I/Cr (159.9 μg/g; IQR: 139.2–189.7 vs. 84.6 μg/g; IQR: 65.3–116.6) were significantly higher in the supplement group. Similarly, the median of estimated 24-h Cr (1.3 g/L; IQR: 1.1–1.4 vs. 1.1 g/L; IQR: 0.94–1.2) and 24-h UIE (205.6 μg/L; IQR: 186.4–225.3 vs. 94.6 μg/L; IQR: 71.4–121.9) were markedly elevated with the use compared with not taking iodine supplements during pregnancy. Moreover, the subgroup utilising iodine supplement had sufficient iodine intake, whereas the women not taking iodine supplements were iodine deficient, as per the WHO cut-off values during pregnancy.

While the use of non-iodised salt (> 3.5-fold) and passive smoking (1.6-fold) were independent factors associated with increased risk of iodine insufficiency during pregnancy, taking iodine supplements was strongly and significantly associated with reduced odds (500-fold) of iodine inadequacy (Table 3). Alternatively, none of the socioeconomic factors increased the risk of iodine intake above requirements during pregnancy, whereas the use of non-iodised salt (6-folds) and 2nd trimester (2.4-fold) were significantly associated with a decreased risk of iodine intake above requirements. Significant independent associations were also detected between the BMI and median UIC in the pregnant population.

**Table 3:** The socioeconomic risk factors associated with insufficient and excess iodine intake among the pregnant participants (*n* = 1222) by multinomial regression analysis

**Risk factors**	**Insufficiency (< 150 μg/L)**		**Above requirements (≥ 250 μg/L)**	
	***Odds ratio (95%CI)***	***P Value***	***Odds ratio (95%CI)***	***P Value***
**Age (years)**	1.007 (0.968–1.048)	NS	0.979 (0.924–1.036)	NS
**BMI (Kg/m**^**2**^**)**	0.951 (0.913–0.991)	***P*** **= 0.01**	1.090 (1.013–1.173)	***P*** **= 0.02**
***Pregnancy groups***				
1st Trimester	1.384 (0.858–2.231)	NS	0.957 (0.481–1.902)	NS
2nd Trimester	1.092 (0.682–1.747)	NS	0.407 (0.175–0.947)	***P*** **= 0.03**
3rd Trimester	**Ref.**		**Ref.**	
***Parity***				
Primiparous	**Ref.**		**Ref.**	
Multiparous	0.862 (0.507–1.464)	NS	1.637 (0.606–4.421)	NS
***Family Size***				
≤ 4 members	**Ref.**		**Ref.**	
> 4 members	0.691 (0.427–1.118)	NS	0.823 (0.406–1.668)	NS
***Education Level***				
Illiterate	0.953 (0.372–2.440)	NS	1.607 (0.596–4.335)	NS
Primary Education	1.487 (0.709–3.117)	NS	1.096 (0.322–3.735)	NS
Secondary Education	0.778 (0.490–1.234)	NS	1.305 (0.605–2.813)	NS
University	**Ref.**		**Ref.**	
***Employment***				
Yes	1.182 (0.716–1.950)	NS	1.082 (0.440–2.659)	NS
No	**Ref.**		Ref.	
***Active smoking***				
Yes	1.996 (0.648–6.144)	NS	0.445 (0.056–3.525)	NS
No	**Ref.**		Ref.	
***Passive smoking***				
Yes	1.653 (1.043–2.618)	***P = 0.03***	1.039 (0.528–2.043)	NS
No	**Ref.**		Ref.	
***Salt Intake***				
Iodized	**Ref.**		**Ref.**	
Non-iodized	3.813 (1.992–7.297)	***P*** **< 0.001**	0.157 (0.036–0.685)	***P*** **= 0.01**
Don’t know	3.444 (1.287–9.214)	***P = 0.01***	1.505 (0.551–4.117)	NS
***Iodine supplement***				
Yes	0.002 (0.001–0.005)	***P < 0.0001***	0.936 (0.478–1.835)	NS
No	**Ref.**			

*Ref* Reference category

*NS* Non-significant

## Discussion

To the best of our knowledge, this study is the first to measure iodine adequacy in reproductive age and pregnant Saudi women. The majority of non-pregnant women (73%) were using iodised salt, 2.8% were taking iodine supplements, and the median UIC was at the lowest WHO limit for iodine adequacy in reproductive age women [13]. In contrast, the pregnant group median UIC was below the WHO minimum for iodine sufficiency despite that iodised salt and/or daily iodine supplements were used by 71.5 and 27.6% of women, respectively. Our findings infer that the reproductive age Saudi women were marginally iodine sufficient, while the pregnant population had mild deficiency that could denote a public health burden [25–28].

The IGN report (2019) has classified the Saudi public as iodine sufficient based on a national SAC survey [5, 16]. Others also showed iodine intake enhancement in SAC from a previously classified severe iodine deficient region after the execution of USI in KSA [33]. Contrariwise, severe iodine deficiency (median UIC 17 μg/L) was reported in 3046 SAC from the same region, and 24% had goitre [25]. Iodine deficiency has also been reported in 1887 SAC (median UIC 84 μg/L) from Makkah province, and 7.4% of them were goitrous [26]. Meanwhile 69% of local salt samples were adequately iodised (15–40 ppm), only 70% of the Saudi households were using iodised salt, which is below the USI target of ≥90% coverage [27]. Our non-pregnant results agree with the studies proclaiming iodine adequacy in the general Saudi public [5, 16, 33]. However, they were at the lowest margin of adequacy, which provides additional sustenance for the demands for banning non-iodised salt in KSA to enhance iodine intake [27].

On the other hand, our pregnant population had mild iodine deficiency at the different trimesters, and it was more pronounced with the non-use of iodine supplements. The daily iodine requirements increase immensely during pregnancy to compensate the physiological increase in iodine metabolism [28]. Hence, iodine deficiency in our pregnant population could be related to inappropriate nutrition since many studies have indicated malnourishment in Saudi pregnant women, and they consumed essential nutrients below the recommended requirements [34–37]. Saudi women from the Western region also had significantly low micronutrients intake, thus their offspring had a higher risk of developing birth defects [38–40]. In agreement, the use of iodine supplements was only confirmed by a minority (27.6%) of our pregnant population. Accordingly, this study reinforces the many calls for improving awareness regarding the importance of iodine intake from dietary and supplement sources during pregnancy [41–44]. Moreover, insufficient iodine during pregnancy may precipitate maternal thyroid disorders alongside poor foetal neurodevelopment [1–4]. We have previously reported that 26.8 and 4.8% of 500 pregnant Saudi women had hypothyroidism and isolated hypothyroxinaemia, respectively [28]. However, little is known about the links between iodine intake and thyroid diseases among pregnant Saudi women. Hence, more studies to measure the associations between iodine intake and thyroid functions in pregnant Saudi women are needed.

Additionally, this study supports the notion that SAC median UIC could imprecisely reflect iodine status in pregnancy [7–11]. In consolidation, The IGN has revealed that 29 countries reported iodine deficiency in pregnant women despite that their SAC were sufficient [45]. The UNICEF guidelines have likewise stated that median UIC may conceal suboptimal iodine intake in pregnant women [17]. Studies from different countries have also demonstrated marked iodine insufficiency among pregnant women despite using iodised salt and/or iodine supplements [7, 9, 11]. Taken together, our study and the prior reports advocate that the health authorities in each country should consider measuring UIC independently from SAC to accurately evaluate iodine intake in pregnant women [7–11]. Educational programs should also be developed to increase the awareness of pregnant women, or those who are planning for conception, about the significance of iodine for them and for their offspring wellbeing [46, 47].

Measuring composite 24-h UIC is the reference biochemical approach for accurately assessing iodine status [13–15]. However, compliance with the collection of 24-h urine is low and, therefore, spot UIC as well as estimating 24-h UIE have been suggested as convenient and accurate alternative methods for measuring iodine intake [13–15]. Our results demonstrated that the spot I/Cr ratio and the estimated 24-h UIE results were comparable in each of the targeted populations, which agree with numerous earlier studies that recommended the use of spot UIC as an alternative reliable method for estimating iodine status [14, 15, 48].

Iodine adequacy could be influenced by numerous factors [49]. Herein, the risk of iodine deficiency in the non-pregnant population increased with multiparity, which agrees with studies from numerous Western countries [50–53]. A possible construal for the associations between parity and iodine intake could be illustrated by the proclaimed cumulative and non-reversible goitrogenic effects associated with each pregnancy, which may require increasing iodine supply in subsequent pregnancies [54]. Additionally, earning below the Saudi minimal wage also increased the odds of inadequate iodine in our non-pregnant women. Likewise, a linkage between poverty and iodine inadequacy has been reported by community studies, which could be due poor adherence of low-income populations to appropriate micronutrients and iodised salt intake [55, 56]. Numerous population-based studies have also demonstrated the negative impact of smoking on thyroid functions and iodine adequacy in reproductive age and pregnant women [49, 51, 52, 57, 58]. In agreement, our data showed that passive smoking was an independent factor that significantly increased the risk of iodine inadequacy in both the non-pregnant and pregnant groups. Accordingly, this study strengthens the numerous requests to employ the necessary policies for smoking cessation as well as to encourage pregnant women to avoid staying in rooms where others have smoked [59].

Our results also revealed higher risks of iodine insufficiency associated with the consumption of non-iodised salt in the reproductive age and pregnant women. The WHO and UNICEF have adopted the USI policy since 1994 to ensure adequate iodine intake by the general public [60]. Although USI is implemented in KSA, the household consumption of iodised salt (70%) was below the targeted 90% usage by the general population to avoid deficiency [27]. Recently, the WHO has also recommended salt reduction (5 g/day) for adults, including reproductive age and pregnant women, to reduce the likelihood of developing cardiovascular diseases [6]. Suggested plans to simultaneously maintain iodine adequacy with decreasing salt intake include fortifying salt with higher amounts of iodine [6]. Alternatively, Australia and New Zealand have adopted another strategy involving fortifying bread with iodine, and several studies have reported adequate median UIC in adults, including pregnant women, post-fortification [61, 62]. Therefore, the inadequate use of iodised salt in KSA alongside the recommended reduction of salt intake accentuate the importance of developing other vehicle(s) for delivering iodine, thus decreasing iodine deficiency disorders [61, 62]. Additionally, the non-pregnant and pregnant women who reported using iodine supplements in our study were iodine sufficient, whereas those who were not using iodine supplements had iodine insufficiency. Hence, reproductive age (150 μg/day) and pregnant (250 μg/day) women could temporarily benefit from using daily iodine supplements till developing a solid and effective national salt/bread iodisation program [27, 61, 62].

Herein, a weak positive association between BMI and UIC was observed, which correlates with previous reports from Bangladesh and Romania [63, 64]. A possible explanation could be that pregnant and non-pregnant women with high BMI were consuming higher foods rich in iodine than lean individuals. Additionally, pregnant women often change their dietary habits and obese women tend to eat more fish and milk, the richest sources of iodine [65]. On the contrary, others have reported either negative association between BMI and UIC [9, 66] or no correlation between body weight and iodine intake [8, 41]. These controversial results could be linked to different eating habits and/or dietary patterns between the studied populations [67, 68].

The present study has several limitations. Although the number of participants is larger compared with several other reports on pregnant women [7–9], our participants were enrolled from a single site, and other cities from the same region were not included. Additionally, we did not measure the dietary habits and nutritional intake alongside the thyroid function parameters to investigate their correlations with iodine status. However, this is a phase 1 study and we will conduct further research to measure the interactions between nutritional habits, iodine intake and thyroid functions in pregnant women.

## Conclusions

This is the first study to demonstrated borderline iodine sufficiency among reproductive age women from the Western region of Saudi Arabia, whereas the pregnant women were deficient, despite the use of iodised salt. Similar to other countries, this study highlighted the need to survey iodine intake in pregnant women regularly and independently from schoolchildren. Although the use of iodine supplements was associated with a significant decreased risk of deficiency among the targeted populations, less than 30% of pregnant women reported using supplements rich in iodine. Hence, our findings advocate the necessity to develop/implement effective national programs in the different regions of KSA to overcome any potential complications arising from suboptimal iodine intake during pregnancy. Suggested actions include developing educational and awareness campaigns regarding the importance of iodine, and to encourage adequate nutritional schemes based on diets rich in iodine (e.g. fish, milk, iodised salt intake and iodine fortified foods). However, further studies are mandatory to assess the factual magnitude as well as the potential complications of iodine deficiency during pregnancy in the kingdom.

## Supplementary information

**Additional file 1.**

**Additional file 2: Table S1.** The demographic and socioeconomic characteristics of the non-pregnant participants (*n* = 400).

## Data Availability

All data generated or analysed during this study are included in this published article and its supplementary information files (Supplementary files 1 and 2).

## Abbreviations

24-h Cr: 24-h urine creatinine excretion
24-h UIE: Estimated 24-h urine iodine excretion
BMI: Body mass index
KSA: Kingdom of Saudi Arabia
I/Cr: Iodine/creatinine ratio
IGN: Iodine Global Network
IQR: Interquartile range
KIO_3_: Potassium iodate
SAC: School-age children
SAR: Saudi Riyal
SD: Standard deviation
UIC: Urine iodine concentration
USI: Universal salt iodisation
WHO: World Health Organisation
