# Supplementary material for: Iodine adequacy in reproductive age and pregnant women living in the Western region of Saudi Arabia
Source: BMC Pregnancy Childbirth. 2020 Jun 22;20:370. doi: 10.1186/s12884-020-03057-w (PMC7310473; doi:10.1186/s12884-020-03057-w)
Supplement: Supplementary file 2 — Additional file 2: Table S1. The demographic and socioeconomic characteristics of the non-pregnant participants (n = 400). [file 12884_2020_3057_MOESM2_ESM.docx]

**Supplementary Table 1:** The demographic and socioeconomic characteristics of the non-pregnant participants (n = 400).

| **Parameter** | **Non-pregnant**  **(n = 400)** | ***P-value*** |
| --- | --- | --- |
| ***Mean ± SD of Age (year)*** | 29.1 ± 7.3 | **N/A** |
| ***Age groups***  18- <25  25- <35  >35 | 154 (9.5%)  140 (8.6%)  106 (6.5%) | **< 0.01** |
| ***Mean ± SD of Weight (kg)*** | 60.4 ± 13.7 | **N/A** |
| ***Mean ± SD of Height (cm)*** | 158.5 ± 6.3 | **N/A** |
| ***Mean ± SD of BMI (kg/m^2^)*** | 24.03 ± 5.4 | **N/A** |
| ***BMI Classes***  Underweight  Normal  Overweight  Obese | 58 (3.6%)  190 (11.7%)  97 (6.0%)  55 (3.4%) | **< 0.001** |
| ***Parity***  Primiparous  Multiparous | 228 (14.1%)  172 (10.6%) | **0.005** |
| ***Family Size***  ≤ 4 members  > 4 members | 236 (14.6%)  164 (10.1%) | **<0.001** |
| ***Total income (SR)***  < 3000  3001-5,000  5,001-10,000  > 10,001 | 56 (3.4%)  149 (9.2%)  134 (8.3%)  61 (3.8%) | **< 0.001** |
| ***Education Level***  Illiterate  1^ry^ Education  2^ry^ Education  University | 19 (1.2%)  34 (2.1%)  125 (7.7%)  222 (13.7%) | **< 0.001** |
| ***Employment***  Yes  No | 89 (5.5%)  311 (19.2%) | **<0.001** |
| ***Residency***  Urban  Rural | 393 (24.3%)  7 (0.4%) | **<0.001** |
| ***Active smoking***  Yes  No | 23 (1.4%)  377 (23.3%) | **<0.001** |
| ***Passive smoking***  Yes  No | 137 (8.5%)  263 (16.2%) | **<0.001** |
| ***Salt Intake***  Don’t know  Non-iodised  Iodised | 24 (1.5%)  84 (5.2%)  292 (18%) | **<0.001** |
| ***Iodine supplement***  Yes  No | 46 (2.8%)  354 (21.8%) | **< 0.001** |

^N/A = Not applicable^
